# Molecular Variation at the *SLC6A3* Locus Predicts Lifetime Risk of PTSD in the Detroit Neighborhood Health Study

**DOI:** 10.1371/journal.pone.0039184

**Published:** 2012-06-26

**Authors:** Shun-Chiao Chang, Karestan C. Koenen, Sandro Galea, Allison E. Aiello, Richelo Soliven, Derek E. Wildman, Monica Uddin

**Affiliations:** 1 Department of Society, Human Development, and Health, Harvard School of Public Health, Boston, Massachusetts, United States of America; 2 Department of Epidemiology, Mailman School of Public Health, Columbia University, New York, New York, United States of America; 3 Department of Epidemiology, University of Michigan School of Public Health, Ann Arbor, Michigan, United States of America; 4 Center for Molecular Medicine and Genetics, Wayne State University School of Medicine, Detroit, Michigan, United States of America; 5 Department of Psychiatry and Behavioral Neurosciences, Wayne State University School of Medicine, Detroit, Michigan, United States of America; The University of Texas M. D. Anderson Cancer Center, United States of America

## Abstract

Recent work suggests that the 9-repeat (9R) allele located in the 3′UTR VNTR of the *SLC6A3* gene increases risk of posttraumatic stress disorder (PTSD). However, no study reporting this association to date has been based on population-based samples. Furthermore, no study of which we are aware has assessed the joint action of genetic and DNA methylation variation at *SLC6A3* on risk of PTSD. In this study, we assessed whether molecular variation at *SLC6A3* locus influences risk of PTSD. Participants (n = 320; 62 cases/258 controls) were drawn from an urban, community-based sample of predominantly African American Detroit adult residents, and included those who had completed a baseline telephone survey, had provided blood specimens, and had a homozygous genotype for either the 9R or 10R allele or a heterozygous 9R/10R genotype. The influence of DNA methylation variation in the *SLC6A3* promoter locus was also assessed in a subset of participants with available methylation data (n = 83; 16 cases/67 controls). In the full analytic sample, 9R allele carriers had almost double the risk of lifetime PTSD compared to 10R/10R genotype carriers (OR = 1.98, 95% CI = 1.02–3.86), controlling for age, sex, race, socioeconomic status, number of traumas, smoking, and lifetime depression. In the subsample of participants with available methylation data, a significant (*p* = 0.008) interaction was observed whereby 9R allele carriers showed an increased risk of lifetime PTSD only in conjunction with high methylation in the *SLC6A3* promoter locus, controlling for the same covariates. Our results confirm previous reports supporting a role for the 9R allele in increasing susceptibility to PTSD. They further extend these findings by providing preliminary evidence that a “double hit” model, including both a putatively reduced-function allele and high methylation in the promoter region, may more accurately capture molecular risk of PTSD at the *SLC6A3* locus.

## Introduction

Posttraumatic stress disorder (PTSD) is a complex disorder characterized by three symptom clusters including re-experiencing, avoidance, and hyperarousal [1]. Twin studies have shown that genetic influences account for a substantial proportion (35–70%) of variance in PTSD risk [2]–[4]. However, the molecular and genetic basis of this inherited liability is still largely unknown.

The *SLC6A3* (solute carrier family 6 (neurotransmitter transporter, dopamine), member 3; also known as *DAT1* or *DAT)* locus is a biologically plausible candidate gene for PTSD. *SLC6A3* encodes a dopamine transporter, a member of the sodium- and chloride-dependent neurotransmitter transporter family, which plays a key role in the regulation of dopaminergic neurotransmission by removing dopamine from the synaptic cleft via reuptake through the transporter [5]. The role of dopamine in the etiology of PTSD is supported by findings of elevated urinary [6] and plasma [7] levels of dopamine among those affected by the disorder, and by reports of a significant correlation between dopamine concentration and severity of PTSD symptoms in affected individuals [6]. Nevertheless, studies investigating the association between genetic variation at the *SLC6A3* locus and PTSD have produced conflicting results.

The *SLC6A3* locus is characterized by a 40-base-pair variable number tandem repeat (VNTR) polymorphism in its 3′-untranslated region (UTR) which can be present in 3 to 11 copies [8]. Across most populations [9], including African-americans, [9], [10] the 10-repeat (10R) allele is the most frequent followed by the 9-repeat (9R) allele. The VNTR polymorphism has been shown to have a functional effect on *SLC6A3* gene expression; however, some studies indicate that 10R alleles enhance the *SLC6A3* gene expression compared to 9R alleles whereas others indicate the opposite [11]–[18]. Similarly, results investigating the influence of the *SLC6A3* VNTR polymorphism on PTSD risk have been equivocal, with, for example, the 9R allele related to an increased risk of PTSD and/or hypervigilance symptoms in three studies [19]–[21], but not in an additional, recent multigenerational study of families exposed to a natural disaster [22].

The complexity of the association between *SLC6A3* and PTSD may be related, in part, to emerging evidence that not only genetic, but also epigenetic factors shape risk of mental illness. Epigenetic dysregulation has been implicated in pathogenesis of several psychiatric disorders such as depression [23], schizophrenia [24], eating disorders [25], and PTSD [26], [27]. However, little is known about the epigenetic processes regulating *SLC6A3*, and the role of *SLC6A3* methylation in PTSD has, to our knowledge, not yet been reported. To better elucidate the molecular basis shaping risk of PTSD at the *SLC6A3* locus, here we investigate whether the 9R or 10R allele is associated with lifetime PTSD. Using specimens drawn from a population-based cohort, the Detroit Neighborhood Health Study, we assess this putative PTSD-associated genetic variation in one of the largest datasets reported to date. We further conduct an exploratory, pilot investigation of interacting genetic and epigenetic *SLC6A3* variation shaping risk of PTSD using a subset of individuals from our larger genetic dataset.

## Materials and Methods

### Subjects and Ethics Statement

The Detroit Neighborhood Health Study (DNHS) recruited 1,547 adults aged 18 years or older at baseline from the city of Detroit. Data for this study were obtained from consenting participants during this baseline survey year. At wave 1 (baseline), lifetime trauma exposure and PTSD were assessed using structured telephone interviews, and each participant received $25 for their participation in the survey. All survey participants were offered the opportunity to provide venipuncture (VP) blood specimens for the biospecimen component of the study (which included testing of immune and inflammatory markers from serum as well as genetic testing of DNA) and received an additional $25 if they elected to do so. VP specimens were obtained via written, informed consent from a subsample of eligible participants during wave 1 (n = 501). The DNHS was approved by the Institutional Review Board at the University of Michigan (HUM00014138; FWA00004969; OHRP IRB IRB00000245). More details regarding the DNHS can be found in [27].

The original sample for this study consisted of 394 individuals, who were randomly selected from the consenting participants of the blood draw, blinded to their PTSD status. Because the diagnosis of PTSD requires a triggering trauma in order to be expressed, we further restricted our analysis to 362 people who had experienced one or more traumatic events. The high prevalence of lifetime trauma exposure in this genotyped sample (91.9%) is consistent with the prevalence of the full DNHS survey sample [28] and with earlier work focused on adults in the Metro Detroit area [29]. Due to the low frequency of 3′UTR VNTR polymorphism of *SLC6A3* other than 9R and 10R, only the individuals carrying 9R/9R, 9R/10R, or 10R/10R genotypes (n = 320; 62 PTSD cases and 258 non-PTSD controls) were included in the final analysis. The *SLC6A3* gene-methylation interaction was tested in a pilot sample of 83 individuals (16 cases/67 controls) who also had DNA methylation data.

### Assessment of Post-traumatic Stress Disorder and Other Survey-based Variables

Lifetime PTSD was assessed via telephone interview using a modified version of the PTSD Checklist (PCL-C) [30], with additional questions about duration, timing, and impairment or disability due to the symptoms in order to identify PTSD cases that were compatible with DSM-IV criteria. Participants were asked to identify traumatic events they had experienced in the past from a list of 19 specific events [29], and one additional question that allowed participants to briefly describe any other stressful event. Participants who reported experiencing more than one traumatic event were asked to select one event they considered to be the worst and report the posttraumatic symptoms due to that specific event. If participants had experienced more than one trauma, they were also asked symptoms based on a randomly chosen traumatic event from the remaining traumatic events. Respondents were considered affected by lifetime PTSD, if all six DSM-IV criteria were met in reference to either the worst or the random event. The identification of PTSD obtained from the telephone interview responses has been validated in a random subsample of 51 participants via in-person clinical interview, which has been described previously [27], [31]. The comparison showed high internal consistency and concordance.

Additional survey-based variables included in this study were: demographic variables including race, sex, and age; number of traumatic events, known to be strongly associated with PTSD [32]–[34], which was assessed as a count of the different types of traumatic events and ranged from 0–19 for each person; whether a participant had ever smoked, due to the known influence of smoking on DNA methylation levels [35]; socioeconomic position (SEP); and lifetime depression, a mental illness frequently comorbid with PTSD [36]. Consistent with the evidence that attainment of more than a high school education is associated with improved health [37], analyses were performed with SEP dichotomized according to more than high school (high SEP) or high school or less (low SEP). Assessment of the presence/absence of lifetime depression in the DNHS has been previously reported in detail, and has been validated via clinical in-person interviews [38].

### Genotyping

Samples were genotyped for the *SLC6A3* 3′UTR VNTR using the primer sets described in Drury et al [19]. Genotyping was performed on the Mastercycler Pro S thermocycler (Eppendorf, Hamburg, Germany), using Qiagen©’s Taq PCR Core Kit and associated protocols. Thermocycling conditions included a 94°C initial at 2 minutes followed by, 35 cycles of: 94°C denature for 15 seconds, 64°C annealing temperature for 15 seconds and a 72°C extension for 30 seconds; and a final temperature of 72°C for 5 minutes. PCR products were then size fractionated on a 2% agarose gel stained with ethidium bromide. Allele identification was based on fragments ranging from 3 repeats to 11 repeats, from known genotypes and sizes standards described in Michelhaugh et al [11]. Amplification and analysis was performed at least twice for each individual.

### DNA Methylation Microarray Data

Methylation microarray data analysed in this study were obtained from the HumanMethylation27 (HM27) DNA BeadChip (Illumina) as previously described [27]. Bisulfite-converted DNA samples were subjected to methylation profiling via the HumanMethylation27 (HM27) DNA BeadChip (Illumina) following the manufacturer’s instructions. Methylation levels were determined for 27,578 CpG dinucleotides spanning 14,495 genes. The resulting data were background normalized using Bead Studio. The validation of the methylation microarray data via pyrosequencing and DNA sequencing of a subset of individuals tested on the original microarray were conducted and has been reported in detail elsewhere [27]. For the purpose of this study, methylation of *SLC6A3* was assessed at two CpG sites represented on the HM27 BeadChip. The first CpG site (cg13202751) occurs approximately 900bp upstream of the *SLC6A3* gene within its putative promoter region. The second CpG site (cg26205131) is located in the first intron of the *SLC6A3* gene, between the upstream 5′-UTR and the downstream start codon (∼1.5 kb).

### Pyrosequencing Validation

Locus-specific pyrosequencing was conducted to validate the methylation data at cg13202751. Pyrosequencing assays were designed and implemented by EpigenDx (Worcester, MA) following the manufacturer’s recommended protocol. Since the microarray and the pyrosequencing methylation data were not normally distributed in our sample, we evaluated the correlation between the two using the Spearman’s rank order correlation test. We observed a moderate but significant correlation between the two variables based on available DNA samples from 69 of the original 83 individuals tested in the microarray analysis (Spearman’s ρ = 0.31, *p* = 0.009).

### Statistical Analysis

Chi-square tests were performed to verify Hardy-Weinberg equilibrium. We calculated means with standard deviations for continuous covariates. For categorical covariates, frequencies and percents were calculated. Bivariate associations were assessed for each of the variables of interest and covariates with respect to lifetime PTSD status. The chi-square test was performed for categorical variable comparisons; for continuous variable comparison, two-sample t-tests were used. *SLC6A3* 3′UTR VNTR genotypes of 9R/9R and 9R/10R were combined into the ‘9R carrier’ category due to a small number of individuals with 9R/9R genotype (n = 15). Logistic regression analysis, adjusting for potential confounders and known predictors of PTSD, including age, sex, socio-economic position, race, smoking, number of traumatic events, and lifetime depression, was used to assess the main effect of *SLC6A3* VNTR polymorphism on the risk of lifetime PTSD, which was coded as a dichotomous variable. Continuous variables including age and number of traumatic events were centered to the mean.

Because the odds ratio estimation in logistic regression could be unreliable (i.e. overestimated) when sample size is not large, the exact logistic test was used when analyzing the pilot sample consisting of those who also had *SLC6A3* microarray methylation data (n = 83) to ensure a valid inference in such situation. The same covariates as in the genotype analysis plus peripheral blood mononuclear cell (PBMC) counts (collected as previously described in [38]), were adjusted for to assess the main and interacting effects of *SLC6A3* genetic and epigenetic variation on lifetime risk of PTSD. In the exact logistic test, all continuous variables were dichotomized by the median value except the total number of PTEs to make the exact test computationally feasible. Due to the limited variation in the methylation beta-values at cg26205131 (Figure S1), our statistical analysis focused on cg13202751. Methylation beta-values at cg13202751were dichotomized based on the median value (median-split; 0.19) to improve the estimation stability of the logistic regression models. In all analyses, *p*-values of less than 0.05 (two-tailed) were considered as evidence of statistical significance. The analyses were conducted with SAS version 9.2 (SAS Institute, Cary, NC).

## Results

### Full Analytic Sample

In Table 1, we present the frequencies of all *SLC6A3* 3′-UTR alleles for all 362 trauma exposed participants. The descriptive statistics and bivariate results based on the 320 trauma exposed study participants with either 9R/9R, 9R/10R, or 10R/10R genotypes are shown in Table 2. The majority of participants (79.3%) were of African American descent. The lifetime prevalence of PTSD in this sample was 19.4%. Compared to individuals without PTSD, PTSD cases reported significantly greater number of traumatic events (*p* < 0.001), were more likely to have ever smoked (p = 0.02), and were more likely to have met lifetime criteria of depression (*p* < 0.001). The *SLC6A3* genotype distribution for participants carrying a 9R or 10R allele did not depart from Hardy-Weinberg equilibrium (*p* = 0.15). After adjusting for age, sex, socio-economic status, race, smoking, number of traumatic events and lifetime depression, 9R allele carriers showed almost twice the risk of PTSD compared to 10R/10R carriers (OR = 1.98, 95% CI = 1.02–3.86) (Table 2).

**Table 1:** Allele frequencies of *SLC6A3* 3′-UTR VNTR polymorphism in trauma-exposed participants (n = 362).

Allele	Frequency	Percent (%)
3R	28	3.93
7R	5	0.70
8R	14	1.97
9R	126	17.70
10R	537	75.42
11R	2	0.28
Missing*	12	–

* 6 individuals failed *SLC6A3* 3-‘UTR VNTR genotyping.

**Table 2:** Descriptive statistics and bivariate comparisons of participants with and without lifetime PTSD and effect of *SLC6A3* 3′UTR VNTR polymorphism on risk of lifetime PTSD in the full analytic sample* (n = 320).

	Overall trauma exposed sample (n = 320)		non-PTSD controls (n = 258)		PTSD cases (N = 62)			Main effect model			
Characteristic	N	%	N	%	N	%	p-value	OR	95% CI of OR		p-value
Age**	51.6	15.76	52.19	16.15	61	49.1	0.17	1	0.98	1.02	0.94
Female	186	58.13	146	56.59	40	64.52	0.26	1.52	0.77	2.96	0.23
Low SES	149	46.56	114	44.19	35	56.45	0.08	2.69	1.37	5.3	**<0.01**
African American	253	79.31	199	77.43	54	87.1	0.09	1.86	0.74	4.69	0.19
Ever smoke	207	64.69	159	61.63	48	77.42	**0.02**	2.19	1.01	4.72	0.05
Lifetime depression	76	23.82	43	16.73	33	53.23	**<0.0001**	5.55	2.74	11.21	**<0.0001**
Number of PTEs**	5.95	3.56	5.42	3.28	8.16	3.83	**<0.0001**	1.19	1.08	1.31	**<0.001**
*SLC6A3* 3′-UTR VNTR genotype											
9R carriers	104	32.5	79	30.62	25	40.32	0.14	1.98	1.02	3.86	**0.04**
10R/10R	216	67.5	179	69.38	37	59.68		1	(referent group)		

SES: socio-economic status; PTEs: potential traumatic events; UTR: untranslated region; VNTR: variable number tandem repeat.

* Full analytic sample includes 320 participants who were trauma exposed and had *SLC6A3* 3′-UTR VNTR polymorphism of either 9R/9R, 9R/10R, or 10R/10R genotypes.

** Variables are presented by mean and standard deviation.

### DNA Methylation Subsample

The lifetime prevalence of PTSD in the DNA methylation subsample was 19.2%. Similar to the full analytic sample, participants with PTSD in the methylation subsample reported a significantly greater number of traumatic events (*p* = 0.001), were more likely to have ever smoked (p = 0.01), and were marginally more likely to have met lifetime criteria of depression (*p* = 0.06) compared to non-PTSD affected participants. Mean DNA methylation beta-values at cg13202751 did not differ significantly by PTSD status (*p* = 0.56).

In main effect analyses (Table 3), there was no significant evidence of association between high methylation level at *SLC6A3* CpG site cg13202751 and lifetime PTSD after adjusting for age, sex, socio-economic position, race, smoking, number of traumatic events, PBMC counts, and lifetime depression (*p* = 0.39). However, results from the exact logistic regression test for lifetime risk of PTSD indicated a significant *SLC6A3* genotype × methylation interaction (*p* = 0.008). Specifically, 9R allele carriers showed an increased risk of lifetime PTSD only in conjunction with high methylation at cg13202751 located within the *SLC6A3* promoter locus.

**Table 3:** Main effects of *SLC6A3* 3′UTR VNTR polymorphism and promoter region methylation and interactive on risk of lifetime PTSD in the methylation subsample (n = 83).

Adjusted Models	Main effect - *SLC6A3* VNTR genotype				Interaction model			
Characteristic	OR	95% CI of OR		p	OR	95% CI of OR		p
Age*	0.47	0.08	2.40	0.49	0.29	0.02	2.24	0.35
Female	1.31	0.24	7.37	1.00	1.13	0.14	9.18	1.00
Low SES	3.25	0.73	18.86	0.15	18.19	1.79	>999.99	**<0.01**
African American	0.55	0.08	4.10	0.74	0.82	0.08	10.77	1.00
Ever smoke	5.37	0.90	63.32	0.07	11.08	0.86	868.49	0.08
PBMC counts*	0.49	0.10	2.11	0.44	0.72	0.13	3.82	0.92
Number of PTEs †	1.25	1.04	1.54	**0.02**	1.44	1.10	2.04	**<0.01**
Lifetime depression	2.29	0.43	13.11	0.43	2.37	0.35	18.29	0.52
*SLC6A3* methylation * ^,^ ‡	2.34	0.47	13.74	0.39	0.31	0.02	3.14	0.47
*SLC6A3* VNTR genotype	1.69	0.34	8.33	0.67	0.05	<0.001	1.46	0.11
*SLC6A3* methylation x genotype interaction	–	–	–	–	48.61*	2.73	Infinity	**<0.01**

SES: socio-economic status; PTEs: potential traumatic events; UTR: untranslated region; VNTR: variable number tandem repeat; PBMC: peripheral blood mononuclear cell counts.

* Median-split.

† Continuous, centered to the mean.

‡ Assessed at cg13202751.

## Discussion

In this work, we have explored how genetic and epigenetic molecular variation at the *SLC6A3* locus shapes risk of PTSD. Our findings confirm previous work indicating that 9R allele carriers of the *SLC6A3* 3′UTR VNTR polymorphism show significantly increased risk of lifetime PTSD compared to 10R/10R genotype carriers [19]–[21]. In addition, we provide preliminary, new evidence that interacting genetic and epigenetic variation at the *SLC6A3* locus shapes risk of PTSD, with participants who carried 9R alleles and possessing high DNA methylation at cg13202751 showing significantly increased risk of the disorder. Although these preliminary findings await confirmation, we suggest that an integrated model that simultaneously investigates the interaction between genetic polymorphisms and epigenetic alterations, as conducted here, may contribute to a more comprehensive picture of the complex molecular etiology shaping risk of PTSD.

Our findings have several implications. First, our results provide indirect support that may help to resolve whether the 9R [11] or 10R *SLC6A3* allele is associated with higher transcription levels [12], [14], [17]. Given the association between elevated dopamine levels and posttraumatic symptoms, discussed in the introduction, our own observation of a significantly increased risk of PTSD in 9R allele carriers suggests that the 9R allele may result in decreased *SLC6A3* transcription, although this cannot be determined with certainty without further functional studies. Second, our results highlight the importance of considering how molecular variation, at multiple levels, can shape risk of complex illnesses like PTSD. Although the relationship between DNA methylation and gene expression is complex, increased promoter-region DNA methylation is typically thought to correlate with decreased gene transcription [39]. Our results identified a significant genotype x methylation interaction, whereby individuals who have the “double hit” risk factors of both a putatively reduced-function 9R allele and high promoter region *SLC6A3* methylation exhibited significantly elevated risk of PTSD. We speculate that these individuals are likely to have elevated dopamine levels in the synaptic cleft that may, in turn, contribute to increased risk of PTSD, but future work in other independent samples is warranted to confirm this initial finding.

The study has several strengths. First, compared to prior studies, this study had a relatively large total sample size. Second, it is the first study that assessed the effect of the *SLC6A3* 3′ UTR VNTR variant on the risk of PTSD in a population-based sample, which reduces potential biases of non-compatibility between cases and controls compared to clinic-based samples or volunteers. Third, no prior studies, to our knowledge, have considered the role of DNA methylation when assessing the involvement of *SLC6A3* in PTSD; similarly, none have considered the joint action of *SLC6A3* genetic and DNA methylation variation on risk of PTSD. This study thus broadens existing knowledge by identifying the ways in which both forms of *SLC6A3* molecular variation shape the risk of PTSD.

Limitations of our study include a relatively small sample size with which to test DNA methylation effects on risk of PTSD; we also note that our results were not corrected for multiple testing in the methylation subsample analyses. In addition, because there are few participants with 3′UTR VNTR homozygous 9R genotypes, we were unable to specifically investigate the effects between homozygous and heterozygous 9R carriers on the risk of PTSD. Furthermore, we were unable to directly assess the relation between 9R vs. 10R alleles on *SLC6A3* gene expression levels as the samples tested in this work were not collected in a manner that preserved RNA. Finally, due to the cross-sectional analysis of blood specimens and questionnaire data, the temporal relationship between *SLC6A3* methylation differences and PTSD onset remain unclear. Ongoing work using samples from this same longitudinal cohort should help to shed light on this issue.

Despite these limitations, results of this study support an important role for the dopamine transporter in PTSD. Our findings are in accordance with studies favoring the 9R allele of the *SLC6A3* 3′UTR VNTR polymorphism as a risk allele for PTSD compared to the homozygous 10R genotype. In addition, to the best of our knowledge, we report the first, albeit preliminary, simultaneous investigation of *SLC6A3* genetic and epigenetic variation on the lifetime risk of PTSD. Individuals had the highest risk of PTSD when they both carried a 9R allele at the 3′UTR VNTR and had showed hypermethylation at a CpG site located in the *SLC6A3*, offering a potential molecular mark of increased risk for PTSD. Future studies conducted on other, independent cohorts should help to confirm the generalizability of our findings.

## Supporting Information

Figure S1 **DNA methylation beta-value distributions of** ***SLC6A3*** **at the two CpG sites (cg13202751 and cg26205131) represented on the HM27 beadchip.**
(TIFF)
