# Supplementary figures and images for: Molecular Variation at the SLC6A3 Locus Predicts Lifetime Risk of PTSD in the Detroit Neighborhood Health Study
Source: PLoS One. 2012 Jun 26;7(6):e39184. doi: 10.1371/journal.pone.0039184 (PMC3383758; doi:10.1371/journal.pone.0039184)

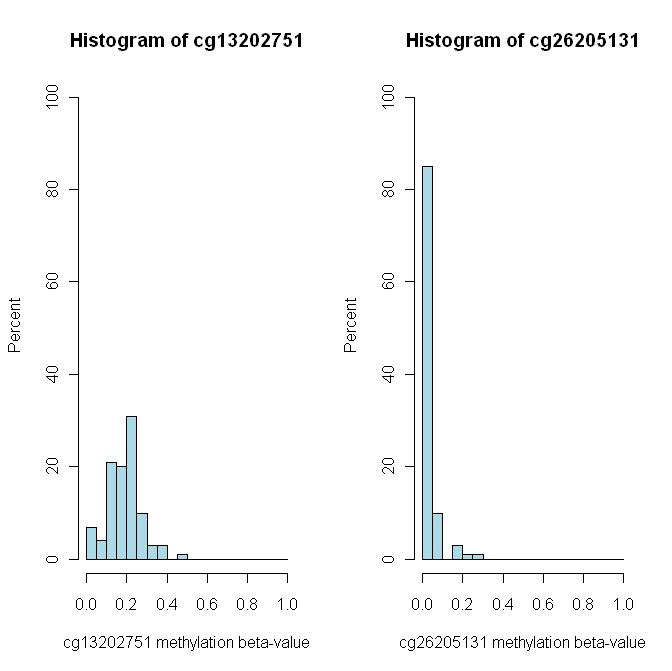

Supplement: Figure S1 — DNA methylation beta-value distributions of SLC6A3 at the two CpG sites (cg13202751 and cg26205131) represented on the HM27 beadchip. (TIFF) [file pone.0039184.s001.tif]
